# Sodium Butyrate Attenuates AGEs-Induced Oxidative Stress and Inflammation by Inhibiting Autophagy and Affecting Cellular Metabolism in THP-1 Cells

**DOI:** 10.3390/molecules27248715

**Published:** 2022-12-09

**Authors:** Man Yan, Xiang Li, Chang Sun, Jiajun Tan, Yuanyuan Liu, Mengqi Li, Zishang Qi, Jiayuan He, Dongxu Wang, Liang Wu

**Affiliations:** 1 Department of Laboratory Medicine, School of Medicine, Jiangsu University, Zhenjiang 212013, China; 2 Medical Laboratory Department, Huai’an Second People’s Hospital, Huai’an 223022, China; 3 Department of Endocrinology, The Affiliated Huai’an No. 1 People’s Hospital of Nanjing Medical University, Huai’an 223300, China; 4 Zhenjiang Center for Disease Control and Prevention, Zhenjiang 212002, China; 5 School of Grain Science and Technology, Jiangsu University of Science and Technology, Zhengjiang 212100, China

**Keywords:** diabetic nephropathy, sodium butyrate, advanced glycation end products, inflammatory damage, cellular metabolism

## Abstract

In recent years, sodium butyrate has gained increased attention for its numerous beneficial properties. However, whether sodium butyrate could alleviate inflammatory damage by macrophage activation and its underlying mechanism remains unclear. The present study used an advanced glycosylation products- (AGEs-) induced inflammatory damage model to study whether sodium butyrate could alleviate oxidative stress, inflammation, and metabolic dysfunction of human monocyte-macrophage originated THP-1 cells in a PI3K-dependent autophagy pathway. The results indicated that sodium butyrate alleviated the AGEs-induced oxidative stress, decreased the level of reactive oxygen species (ROS), increased malondialdehyde (MDA) and mRNA expression of pro-inflammatory cytokines of interleukin (IL)-1β and tumor necrosis factor (TNF)-α, and increased the content of superoxide dismutase (SOD). Sodium butyrate reduced the protein expression of the NLR family, pyrin domain-containing protein 3 (NLRP3) and Caspase-1, and decreased the nucleus expression of nuclear factor-kappaB (NF-κB). Sodium butyrate decreased the expression of light-chain-associated protein B (LC3B) and Beclin-1, and inhibited autophagy. Moreover, sodium butyrate inhibited the activation of the PI3K/Akt pathway in AGEs-induced THP-1 cells. In addition, the metabolomics analysis showed that sodium butyrate could affect the production of phosphatidylcholine, L-glutamic acid, UDP-N-acetylmuraminate, biotinyl-5’-AMP, and other metabolites. In summary, these results revealed that sodium butyrate inhibited autophagy and NLRP3 inflammasome activation by blocking the PI3K/Akt/NF-κB pathway, thereby alleviating oxidative stress, inflammation, and metabolic disorder induced by AGEs.

## 1. Introduction

The excessive activation of macrophages and high oxidative stress in diabetic patients is the important cause of diabetic kidney injury [1,2]; about 25–40% of diabetic patients develop kidney damage that eventually leads to end-stage renal disease, which is one of the main causes of death in patients with diabetes [3]. Due to abnormal glucose metabolism, advanced glycation end products (AGEs) and other glucose toxic products can be produced in diabetic patients through polyol and hexosamine pathways [4,5]. The AGEs can bind to AGEs receptors (receptor for advanced glycation end products, RAGEs) on the surface of macrophages to induce excessive inflammation and oxidative stress [6]. It can promote the migration, activation, and aggregation of macrophages to the kidney, leading to the recruiting of a large number of macrophages in the kidney and eventually leading to irreversible kidney damage [7,8]. Therefore, prevention and management of AGEs-induced macrophage inflammation and oxidative stress are the key to preventing diabetic kidney damage.

The clinical studies have also shown that the increasing dietary fiber intake can effectively improve symptoms and prevent complications in type 2 diabetes (T2DM) [9,10]. Short-chain fatty acids (SCFAs) are produced by the fermentation of indigestible dietary fiber by some anaerobic bacteria in the colon, mainly composed of acetic acid, propionic acid, and butyric acid, and other organic acids with a carbon atom number less than six [11]. A central function of inflammation control is played by microbiota-derived metabolites, specifically SCFAs such as acetate, propionate, and butyrate. As an SCFA, butyrate mainly originates in the gut when dietary fiber ferments; however, it can reach the bloodstream and plays a role in inflammation- and immunity-associated diseases such as inflammatory bowel disease, asthma, and arthritis [12,13,14]. There has been evidence that sodium butyrate (NaB) may be effective in the prevention and treatment of diabetic kidney damage in vivo and in vitro in previous studies [15]. However, the mechanism of NaB ameliorating diabetic kidney damage is unclear; it is speculated that the autophagy and related cell metabolism are a possible signaling pathway [16].

There is a crucial and rudimentary biological process in chronic kidney diseases called autophagy that plays an important role in both physiological and pathological processes [17]. Physiologically, it is the process of degrading proteins and organelles mediated by lysosomes, and regulates cell metabolism and survival. Many studies have shown that a number of signal transduction pathways are involved in the regulation of autophagy, i.e., PI3K/AKT, and the signaling crosstalk between PI3K/AKT and the NOD-like receptor pyrin domain-containing protein 3 (NLRP3) that controls mTOR activity, an important member of the NOD-like receptor (NLR) family, in the cytosol and activates sterile inflammation that was shown to be up-regulated in diabetic patients with kidney injury [18,19,20]. The activation of autophagy may reduce hyperuricemia-induced inflammasome activity by targeting ubiquitylated inflammasomes for degradation and decreasing reactive oxygen species (ROS) production and downstream inflammatory responses [21,22,23]. It is well known that NaB is an important energy substance for intestinal epithelial cells and has the functions of regulating energy metabolism by modulating the gut microbiota. Zhang et al. [24] reported that feeding NaB to insulin-resistant db-/db- mice can improve liver glycogen metabolism. However, whether NaB can alleviate oxidative stress and inflammation through other aspects of cell metabolism remains unclear.

In this study, we investigated the effects of NaB on AGEs-induced inflammatory damage in the human monocyte-macrophage originated THP-1 cells, and autophagy signal pathway and cell metabolism were detected. Our findings elucidated the potential mechanisms of NaB protective function in THP-1 cell inflammation and gave some clues for the potential therapy for diabetic nephropathy.

## 2. Results

### 2.1. NaB Inhibits Cellular Inflammation Induced by AGEs

**Figure 1:**
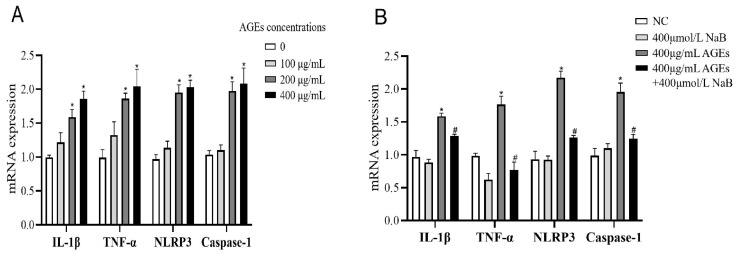
The mRNA expression of inflammatory cytokines of THP-1 cells (*n* = 3/group). (**A**): THP-1 cells were treated with three different concentrations of AGE. (**B**): THP-1 cell were pre-treated with 400 μmol/L of NaB for 24 h, and then treated with 400 μg/mL of AGEs to induce inflammation. NC: normal control; L-AGEs: 100 μg/mL AGEs; M-AGEs: 200 μg/mL AGEs; H-AGEs: 400 μg/mL AGEs; NaB: 400 μmol/L NaB; AGEs: 400 μg/mL AGEs; AGEs+NaB: pre-treated with 400 μmol/L of NaB for 24 h, and then treated with 400 μg/mL of AGEs. *: compared with the NC group, *p* < 0.05; #: compared with the AGEs group, *p* < 0.05.

To determine the ability of AGEs to induce inflammation, THP-1 cells were activated by high, medium, and low concentrations of AGEs (100, 200, and 400 μg/mL), and the expression of inflammatory cytokines was detected by qRT-PCR. The results showed that, compared with the NC group, the mRNA expressions of IL-1β, TNF-α, NLRP3, and Caspase-1 of THP-1 cells were significantly increased when treated with the concentrations of 200 and 400 μg/mL AGEs (Figure 1A). In a subsequent experiment, we used 400 μg/mL AGEs to activate THP-1 cells and 400 μmol/L NaB to inhibit the inflammatory response of THP-1 cells. Compared with the AGEs group, when treated with NaB (400 μmol/L), the mRNA expressions of IL-1β, TNF-α, NLRP3 and Caspase-1 were significantly decreased in the AGEs+NaB group (Figure 1B).

### 2.2. NaB Inhibits Cellular Oxidative Stress Induced by AGEs

**Figure 2:**
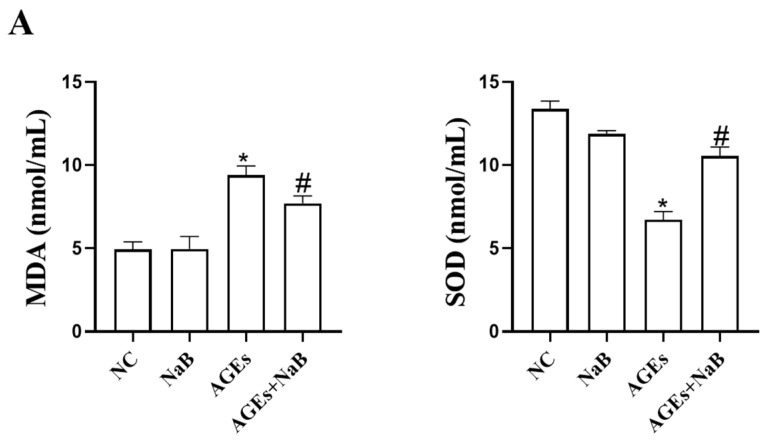

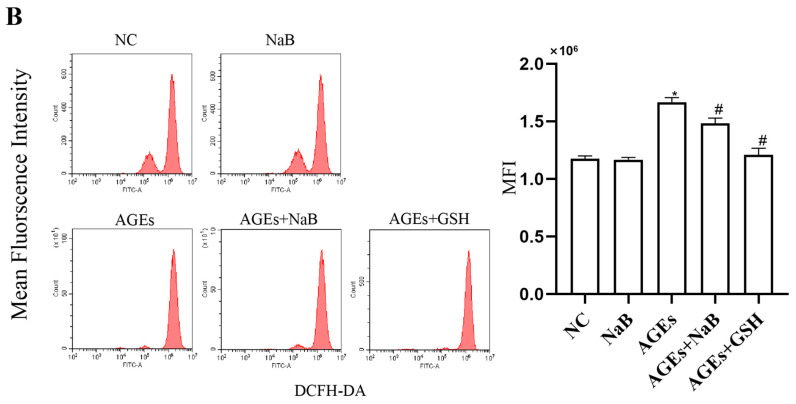
The oxidative stress-related ROS, MDA, and SOD level in THP-1 cells (*n* = 3/group). (**A**): Intracellular MDA level and intracellular SOD level. The MDA level was determined by thibabituric acid (TBA) method. The SOD level was determined by xanthine oxidase method (hydroxylamine method). (**B**): Intracellular ROS level. The intracellular ROS level was determined by DCFH-DA fluorescent probe kit. NC: normal control; NaB: 400 μmol/L NaB; AGEs: 400 μg/mL AGEs; AGEs+NaB: pre-treated with 400 μmol/L of NaB for 24 h, and then treatment with 400 μg/mL of AGEs. *: compared with the NC group, *p* < 0.05; #: compared with the AGEs group, *p* < 0.05.

Oxidative stress plays an important role in the occurrence and development of inflammation. In this study, we determined the concentrations of ROS, MDA, and SOD in THP-1 cells. Compared with the NC group, the concentrations of ROS and MDA in the AGEs group were significantly increased, while the concentration of SOD was significantly decreased (*p* < 0.05) (Figure 2). Compared with the AGEs group, NaB can significantly increase the expression of SOD and decrease the expressions of ROS and MDA (*p* < 0.05) (Figure 2).

### 2.3. NaB Inhibits the Activation of Autophagy Pathway Induced by AGEs

**Figure 3:**
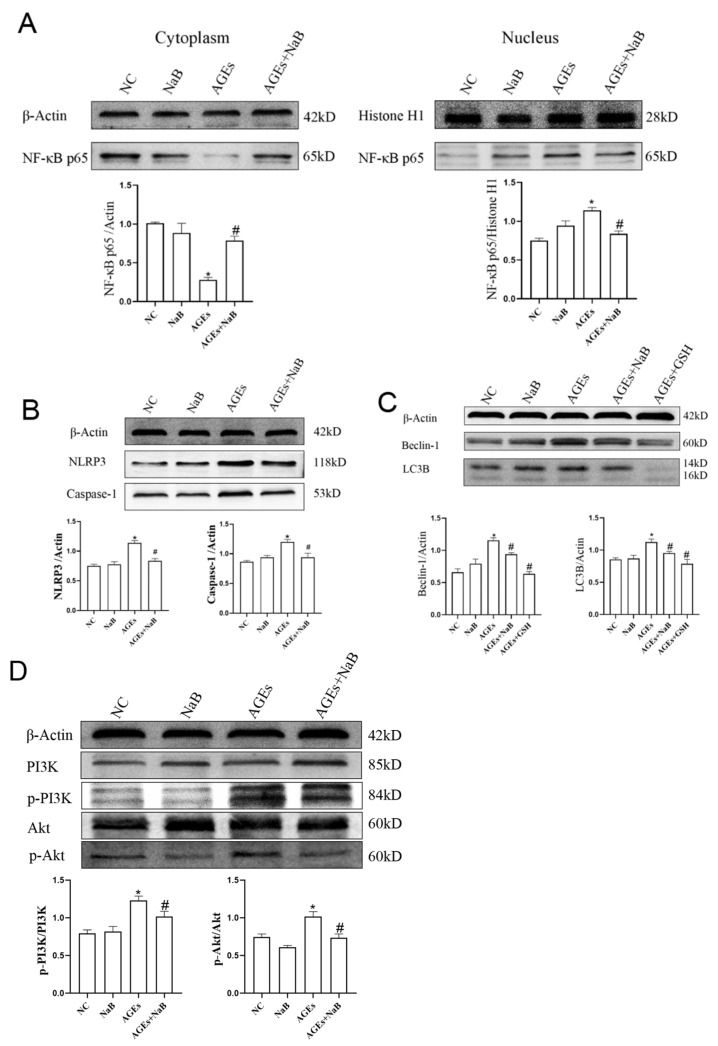
The expression of autophagy-related proteins and inflammation of THP-1 cells (*n* = 3/group). (**A**): The distribution of NF-κB p65 protein in nucleus and cytoplasm of THP-1 cells. (**B**): The expressions of NLRP3 and Caspase-1 associated with inflammation of THP-1 cell. (**C**): The expressions of Beclin-1 and LC3B associated with autophagy of THP-1 cell. (**D**): The expressions of PI3K, p-PI3K, Akt, p-Akt in THP-1 cells. NC: normal control; NaB: 400 μmol/L NaB; AGEs: 400 μg/mL AGEs; AGEs+NaB: pre-treated with 400 μmol/L of NaB for 24 h, and then treated with 400 μg/mL of AGEs; AGEs+GSH: pre-treated with 1 mmol/L of GSH for 1 h, and then treated with 400 μg/mL of AGEs; *: compared with the NC group, *p* < 0.05; #: compared with the AGEs group, *p* < 0.05.

Compared with the AGEs group, after treatment with NaB, the expression of NF-κB p65 in the nucleus was significantly decreased, and the expression of NF-κB p65 in the cytoplasm was significantly increased (*p* < 0.05) (Figure 3A). After treatment with NaB, compared with the AGEs group, the expressions of NLRP3 and Caspase-1 were significantly decreased (*p* < 0.05) (Figure 3B). It is known that beclin-1 and light-chain-associated protein B (LC3B) are key proteins in autophagy, and autophagy plays an important role in regulating inflammation. Compared with the AGEs group, the expressions of Beclin-1 and LC3B in the AGEs+NaB group were significantly decreased (*p* < 0.05) (Figure 3C). The PI3K/Akt pathway is an upstream pathway of NLRP3 and NF-κB and is the important regulatory signal of autophagy. In this study, with the pre-treatment of NaB, the expressions of p-PI3K and p-Akt were significantly inhibited in AGEs-treated cells (*p* < 0.05) (Figure 3D).

### 2.4. NaB Affects the Constitution of Cellular Metabolites

**Figure 4:**
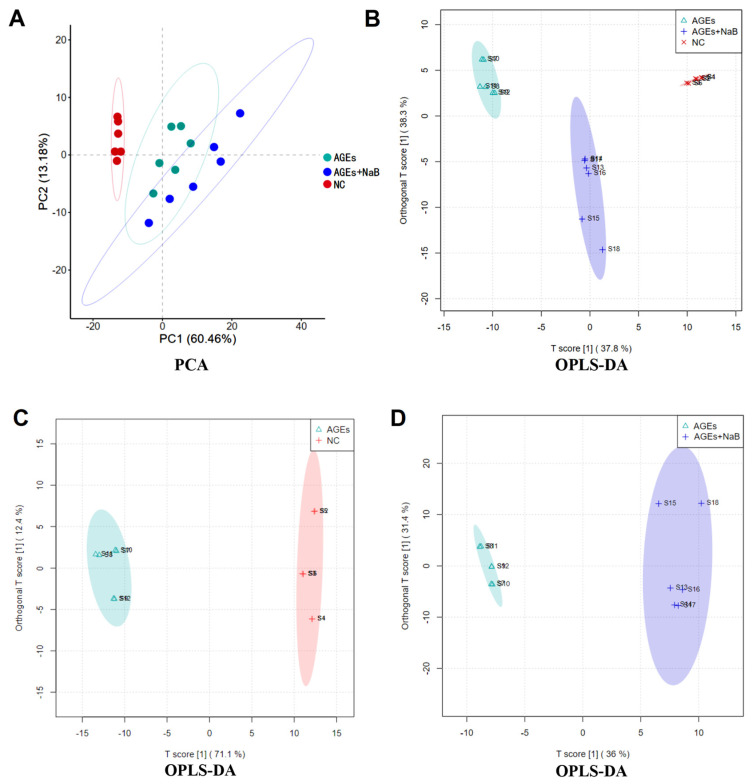
The PCA (**A**) and OPLS-DA (**B**–**D**) score plot. The NC group (red), the AGEs group (green) and the AGEs+NaB group (blue) (*n* = 6/group).

A total of 173 metabolites were detected by comprehensive analysis of the metabolic data of THP-1 cell samples from each group. The principal component analysis (PCA) of these metabolites showed a good clustering among the groups. The NC and AGEs groups could be clearly distinguished, which indicated that the cell metabolites in those two groups above changed significantly when treated with AGEs (Figure 4A). The OPLS-DA model was further used to evaluate the contribution of differential metabolites in THP-1 after AGEs and NaB treatment. In the OPLS-DA score plot, the abscissa was predictive principal component analysis, and the ordinate was orthogonal principal component analysis. The samples of each groups were clearly separated and well-clustered. In this score plot, R2X (the model’s interpretation of X variable) was 0.839, R2Y (the model’s interpretation of Y variable) was 0.989, and Q2 (the model’s predictability) was 0.961. The results showed that there were significant differences in metabolite information among the three groups of samples (Figure 4B).

Next, the differential metabolites among each group were evaluated using variable influence on the project score (VIP > 1) of OPLS-DA and student’s *t*-test analysis (with *p* < 0.05). The variation trends of the above differential metabolites are shown in Table 1. Compared with the NC group, the down-regulated metabolites were L-glutamic acid, ceramide (d18:1/12:0), elaidic acid, and phosphatidylcholine; the up-regulated products were biotinyl-5’-AMP, oxidized glutathione, Prostaglandin F1a (PGF1a), N-methyltryptamine, androsterone, palmitic acid, and 13S-hydroxyoctadecadienoic acid. Compared with the AGEs group, undecanoic acid, PGF1-α, phosphatidylcholine, and L-glutamic acid were significantly up-regulated, and UDP-N-acetylmuraminate, biotinyl-5’-AMP, sphingomyelin (SM) (d18:1/16:0), and thromboxane B2 (TXB2) were significantly down-regulated.

### 2.5. NaB Affects Cellular Metabolic Pathway

**Figure 5:**
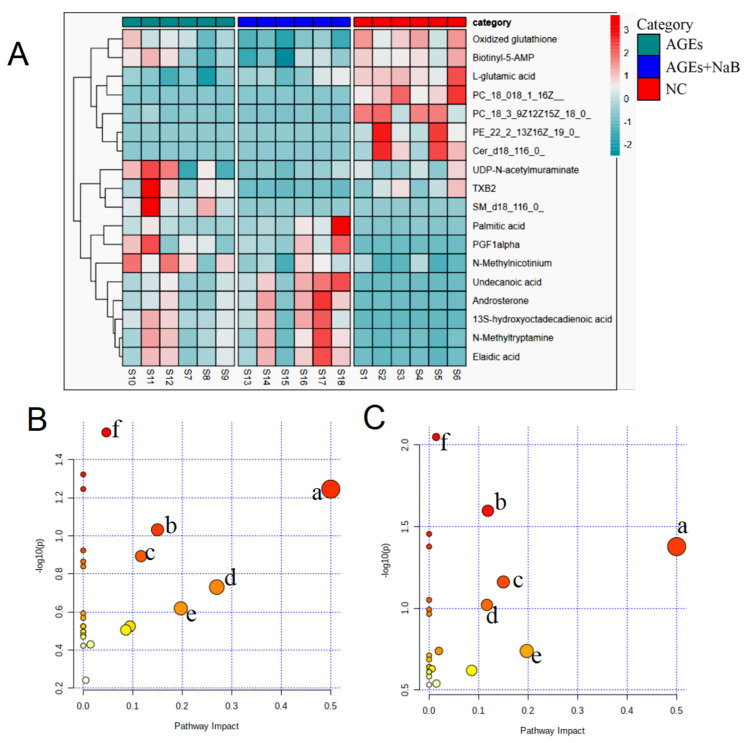
The cluster heat map and metabolic pathway analysis (*n* = 6/group). (**A**): Cluster heat map for the NC group (red), the AGEs group (green), the AGEs+NaB group (blue). (**B**): The metabolic pathway analysis of THP-1 cell metabolites in the NC and AGEs groups. a: D-glutamine and D-glutamate metabolism, b: biotin metabolism, c: arginine biosynthesis, d: sphingolipid metabolism, e: slanine, aspartate and glutamate metabolism, f: glutathione metabolism. (**C**): The metabolic pathway analysis of THP-1 cell metabolites in the AGEs and AGEs+NaB groups. a: D-glutamine and D-glutamate metabolism, b: glycerophospholipid metabolism, c: biotin metabolism, d: arginine biosynthesis metabolism, e: alanine, aspartate and glutamate metabolism, f: sphingolipid metabolism.

The identified differential metabolites were input into the MetaboAnalyst database to construct KEGG analysis of the metabolic pathway, and set the critical value of the influence value of the metabolic pathway as 0.1. If the value was higher than this, it was regarded as a potential target pathway. AGEs treatment could significantly affect multiple metabolic pathways in THP-1 cells, including the D-glutamine and D-glutamate metabolism, biotin metabolism, sphingolipid metabolism, arginine biosynthesis, alanine, aspartate, and glutamate metabolism (Figure 5). The results of the metabolomics study showed that NaB had an excellent therapeutic effect on AGEs-induced metabolic disorders in THP-1 cells.

## 3. Discussion

Gut microbiota plays an important role in the development of T2DM [25]. In fact, the damage of the intestinal mucosal barrier and the long-term chronic inflammation in the body caused by the release of intestinal bacterial endotoxin into the blood through the damaged mucosa can aggravate the insulin resistance and T2DM [26,27]. SCFAs are the products of anaerobic fermentation of undigested carbohydrates in the colon. The concentrations of SCFAs in the intestine were as high as 70–140 mmol/L, and they were easily absorbed into the blood, which can affect the immune and inflammatory response of tissues through the peripheral circulation system [28]. SCFAs can exert anti-inflammatory effects by inhibiting the recruitment and activation of white blood cells and the secretion of various pro-inflammatory cytokines, enhancing the cells’ phagocytic ability. SCFAs are a potential regulator of the “gut-kidney axis”, which plays an anti-inflammatory function in the kidney, among which NaB has the most significant anti-inflammatory effect [29]. Studies have shown that dietary supplements with increased dietary fiber intake, and NaB supplementation can prevent insulin resistance and improve diabetic kidney injury in patients with diabetes, but the specific mechanism needs to be further studied [30].

Diabetic nephropathy is one of the T2DM complications, mainly manifested as glomerular filtration and ultrastructure damage, glomerular basement membrane thickening, and mesangial cell proliferation [31]. In a state of hyperglycemia, protein combines with excess glucose to form a large number of AGEs that are not easily cleared. AGEs can damage renal structure and function by direct cross-linking and binding to macromolecular substances such as proteins, lipids, and nucleic acids [32,33]. In addition, AGEs can interact with the RAGE on the surface of monocytes-macrophages, microglia, and renal innate cells to trigger oxidative stress and produce a large number of ROS [34]. These ROS can increase the concentration of cellular lipid peroxide MDA and lead to the activation of the inflammatory response, which promotes macrophage recruitment and aggravating kidney injury [35]. The results of this study showed that the levels of ROS and MDA were significantly increased in the AGEs treatment group, confirming that AGEs can lead to high levels of oxidative stress response in macrophages. When treated with NaB, the productions of MDA and SOD were significantly decreased. These results suggest that NaB may improve diabetes nephropathy by interfering with inflammatory response.

It is true that inflammation is a protective reaction made by the body, but excessive inflammatory reactions can be harmful. A large number of inflammatory factors produced by the body are the important causes of diabetic kidney injury. Protein kinase B (Akt) is a serine/threonine-specific protein kinase that plays an important biological function in glucose metabolism related to cell apoptosis, survival, and proliferation. Previous studies showed that the hyperglycemic state was able to cause Akt phosphorylation and activate the PI3K/Akt pathway [36]. Meanwhile, the activated Akt can up-regulate IKB-α phosphorylation and NF-κB signaling activation. NF-κB signaling is central to the inflammatory response, activating the NLRP3 inflammasome and subsequently increasing the expression of pro-inflammatory cytokines by macrophages [37]. In this study, we found that the expressions of pro-inflammatory cytokines of IL-1β and TNF-α in the AGEs group were significantly increased, while the expressions of IL-1β and TNF-α were suppressed when treated with NaB.

Autophagy is a special protective phenomenon for mammalian eukaryotic cells to maintain their internal environment. Autophagy can wrap damaged organelles and excessive biological macromolecules in specific membrane structures and send them to lysosomes for degradation and reuse [38]. Autophagy is regulated by a variety of intracellular stimulatory signals, such as oxidative stress, inflammation, and nutritional status. When cells were stimulated by an external environment such as starvation, hypoxia, and oxidative stress, autophagy would be enhanced to maintain the stability of internal environment [39]. LC3B is a hallmark protein on the autophagosome membrane, and the concentration of Beclin-1 reflects the degree of autophagy. It was shown that ROS could induce cellular autophagy [40], and we found the AGEs could also induce the autophagy in THP-1 cells. Our hypothesis was that AGEs induce autophagy as a result of increased intracellular oxidative stress. It was known that moderate autophagy could inhibit inflammation and the expressions of pro-inflammatory cytokines [41]. However, excessive autophagy would cause a large amount of normal cell death and organ function loss [42]. In addition, our results showed that AGEs can also up-regulate the expressions of p-PI3K, p-Akt, NLRP3, and Caspase-1, and enhance the entry of NF-κB p65 protein into the nucleus. However, NaB can reverse the effect of AGEs on THP-1 cells. The results of this study suggested that NaB might inhibit autophagy by inhibiting the intracellular PI3K/Akt pathway.

Glycerol phospholipids are the main substances in cell membrane phospholipids, accounting for about 60% of lipid molecules [43]. Both phosphatidylcholine and phosphatidylthanolamine are metabolites of glycerol phospholipids, which are mainly involved in glycerol phospholipid metabolism [44]. Phospholipids such as Lyso-phosphatidylcholine, phosphatidylcholine, and sphingomyelin have turned out to be associated with insulin resistance and diabetes [45]. A high concentration of AGEs in diabetic patients can activate various protein kinases and increase lipoprotein-associated phospholipase A2 activity. As a result, the degradation of phospholipid compounds in the body is accelerated, leading to a decrease in phospholipid levels in the body. Increasing the number of SCFAs-producing bacteria in the intestine can increase the concentration of phosphatidylcholine precursor and phosphatidylcholine [46]. In this study, the concentration of phosphatidylcholine in THP-1 cells was significantly increased after pre-incubation with NaB, which may be related to glycerol and phospholipid metabolism.

Glutamic acid is the most abundant amino acid in the human body, which provides energy for cell metabolism. It is also the hub of the transformation of purine, pyrimidine, amino sugar, and other amino acids in the body [47,48]. In addition to this, glutamine also participates in the immune response [49,50]. Glutamine could enhance macrophage phagocytosis and immune response by providing ATP for macrophages. Inhibition of glutamine metabolism in macrophages can lead to the transformation of macrophages into M1 type and up-regulate the secretion of pro-inflammatory cytokines, while glutamine supplementation can promote the polarization of macrophages into M2 type and play an anti-inflammatory role [51]. Uridine diphospho-N-acetylglucosamine (UDP-N-acetylglucosamine) is a donor substrate for nucleoplasmic proteins modified with serine and threonine residues by N-acetylglucosamine (O-GlcNAc), which is the final product of the hexosamine biosynthesis pathway. The activation of the hexosamine pathway leads to the production of uridine dipho-sphate N-acetylglucosamine (UDP-GlcNAc) from glucose and glutamine, in which acetyl-CoA and UTP are consumed [52], and O-GlCNAc acetylation promotes inflammation [53]. In this study, the intervention with NaB had a callback effect on glutamine and reduced the concentration of UDP-GlcNAc. After the treatment of NaB, the concentration of glutamine increased significantly while the concentration of UDP-N-acetylmuraminate was decreased. The results indicated that NaB suppressed AGEs-induced inflammation via up-regulating glutamine concentration and glutamic acid metabolism, while down-regulating the concentration of UDP-N-acetylglucosamine. In addition, the relationship between the metabolic changes of biotin and biotinyladenylate and autophagy and inflammation also needs further study.

The present study has several limitations that need to be addressed in additional studies. (1) The beneficial effect of sodium butyrate on AGEs-induced diabetes rodent models has not been investigated, which requires further research. (2) According to our results, the oxidized GSH was reduced in AGEs-treated cells compared with NC cells, and there is a belief that it is related to the self-protective effect of cells at the early stage, but the exact mechanism remains to be determined. (3) Pharmacological inhibition of vacuolar-type H+-ATPase by bafilomycin A1 can regulate autophagy, and further investigation is needed to determine whether bafilomycin A1 affects sodium butyrate’s autophagy inhibition effect.

## 4. Materials and Methods

### 4.1. Cell Culture and Treatment

THP-1 cells were purchased from Shanghai Cell Bank and cultured in RPMI 1640 medium (Biological Industries, Kibbutz, Israel) containing 10% fetal bovine serum (Biological Industries, Kibbutz, Israel) at 37 °C and 5% CO_2_. THP-1 cells were stably cultured to the fourth generation and then seeded into 6-well plates with 1 × 10^6^ cells per well. In this experiment, the cells were divided into 4 groups, including the normal control group (NC group), the sodium butyrate group (NaB group), the advanced glycation end products group (AGEs group), the AGEs+NaB group (AGEs+NaB group). In this experiment, THP-1 cells were induced to macrophage formation with the concentration of 100 ng/mL phorbol-12-myristate-13-acetate (PMA). The PMA was added into the cell culture medium. After 24 h, the NC, NaB, and AGEs groups underwent follow-up treatment, respectively, while the AGEs+NaB group was added with PMA and NaB at the beginning of the experiment, and then added with AGEs 24 h later. The NaB and the AGEs+NaB groups were pre-treated with 400 μmol/L of NaB for 24 h, and then the AGEs+NaB group was treated with 400 μg/mL of AGEs for another 24 h. The AGEs groups were only treated with 400 μg/mL of AGEs for 24 h. The cells in the NC group were cultured normally without any treatment. The cells of each group were collected at the end of the experiment for follow-up study.

### 4.2. Real-Time Quantitative PCR (qRT-PCR)

Total RNA of THP-1 cells was extracted by Trizol method (Vazyme Biotechnology, Nanjing, China) and reverse transcribed into cDNA (Vazyme Biotechnology, China) for qRT-PCR detection (CFX96, Bio-Rad, Hercules, CA, USA). The total PCR reaction system included 10 μL SYBR Green Master premix (Vazyme Biotechnology, China), 0.4 μL (10 μmol/L) of upper and lower primers, and 2 μL cDNA template. The reaction process included initial denaturation at 95 °C for 5 min, denaturation at 95 °C for 3 s, annealing at 58 °C for the 20 s, and extension at 72 °C for 30 s, with a total of 40 cycles. Using GAPDH as an internal reference, the relative gene expression was calculated by formula 2-ΔΔCt. The PCR primers’ sequences are shown in supplementary Table S1.

### 4.3. Western Blotting Analysis

Total cellular proteins were extracted and performed as follows. Collected all the cells and washed twice with precooled phosphate buffer. The proteins were extracted by RIPA lysate buffer (Beyotime Biotechnology, Shanghai, China) and boiled with 5 × SDS-PAGE loading buffer for 10 min. The nucleus and cytoplasmic proteins were isolated strictly according to the instructions (Beyotime Biotechnology, China). All samples were subjected to SDS-PAGE electrophoresis, transferred to PVDF membranes (0.45 μm, Thermo Fisher Scientific, Waltham, MA, USA), then blocked with 5% skim milk for 1 h at room temperature. The rabbit antibodies including NLRP3 (BA3677, 1:250, Boster, Wuhan, China), Caspase-1 (Catalog No.: A16792, 1:1000, Abclone, Wuhan, China), Beclin-1 (A7353, 1:500, Boster, China), LC3B (Catalog No.: A19665, 1:500, Abclone, China), NF-κB p65 (Catalog No.: A10609, 1:500, Abclone, China), Histone H1 (Catalog No.: A4342, 1:500, Abclone, China), PI3K (Cat.#:AF6241, 1:1000, Affinity, China), p-PI3K (Cat.#:AF3242, 1:1000, Affinity, China), Akt (WL0003b, 1:1000, Wanlei, China), p-Akt (WLP001a, 1:1000, Wanlei, China) and β-actin (Catalog No: BA2305, 1:5000, Boster, China) were diluted with TBST buffer and incubated at 4 °C overnight. The PVDF membranes were washed 3 times in TBST buffer and incubated with the goat anti-rabbit IgG-HRP (1:10000, Boster, China) for 1 h. The ECL chemiluminescence kit (Millipore, Boston, MA, USA) was used, and the Image J software was used to analyze and calculate the expression of target proteins. All experiments were repeated 3 times.

### 4.4. The Cell Oxidative Stress Related Factors Detection

The DCFH-DA fluorescent probe kit (Beyotime Biotechnology, Shanghai, China) was used in this experiment to detect the intracellular ROS concentration. The superoxide dismutase (SOD) and malondialdehyde (MDA) kits were produced by Jiancheng Bioengineering Institute (Nanjing, China). The collected THP-1 cells were crushed in a mortar without nuclease, centrifuged at 16,400× *g* for 10 min, and the precipitation was discarded. The supernatant was used to detect the activities of SOD and MDA. In ROS detection, the DCFH-DA probe was diluted with serum-free RPMI 1640 medium at 1:1000. All the cells were suspended in the diluted DCFH-DA probe suspension and incubated for 20 min at 37 °C and mixed every 5 min to make sure the probe was in full contact with the cell. Finally, the serum-free medium was used to wash the cells 3 times to remove the DCFH-DA probe that did not bind to the cells, and suspended with sterile PBS buffer. According to the kit instructions, intracellular ROS was detected by Flow Cytometer (BD Biosciences, Franklin Lake, NJ, USA). In addition, the glutathione (GSH) group (AGEs+GSH group) was set in this experiment. GSH is a commonly used ROS clearance agent. In this study, the final concentration of GSH was 1 mM and the acting time was 1 h. DCFH-DA fluorescent probe kit was used to detect fluorescent removal of ROS induced by AGEs with GSH. All experiments were repeated 3 times.

### 4.5. Cell Collection and Preparation

Collected all the cells and added 2 mL of precooled 80% (*v*/*v*) methanol, placed at −80 °C for 20 min, then incubated at −20 °C for 1 h after ultrasonic treatment for 10 min. Then centrifuged at 4 °C at 16,400× *g* for 5 min. Collected the supernatant and dried in a freeze dryer. The lyophilized cell extract was redissolved with 200 μL 50% acetonitrile aqueous solution and fully dissolved by ultrasonic treatment for 10 min. After centrifugation at 4 °C and 16,400× *g* for 15 min, the supernatant was taken for subsequent analysis. The quality control sample consisted of 10 μL of each sample.

### 4.6. Chromatography and Mass Spectrometry Conditions

The chromatographic experiment was performed on an ACQUITY UPLC BEH C18 column (Waters, Milford, MA, USA, 100 mm × 2.1 mm, 1.7 μm) in the ultra-high-performance liquid chromatography (UPLC) system (Waters, USA). The column temperature was maintained at 30 °C. The flow rate was set at 0.2 mL/min. The sample injection volume was 5 µL. Solvent A was water mixed with 0.1% formic acid, and solvent B was acetonitrile. The gradient elution programs were: 0–2 min, 1–1%B; 2–8 min, 1–35%B; 8–11 min, 35–40%B; 11–14 min, 40–60%B; 14–17 min, 60–99%B; 17–18 min, 99–99%B; 18–19 min, 99–1%B; 19–20 min, 1–1%B. Electrospray ionization source (ESI) was used in high-resolution quadrupole mass spectrometer. Used ESI electrospray ionization method, and positive and negative ion mode was used for acquisition. The flow and temperature of desolvation gas were 700 L/h and 320 °C, respectively. The flow rate of the cone back flush gas was 50 L/h. The capillary voltage was 3 kV; cone hole voltage was 40 V; and source deviation voltage was 80 V. The mass scan range was 50~1500 Da.

### 4.7. Multivariate Data Processing and Data Processing

Peak recognition, peak matching, peak alignment, and normalization were performed on the raw data UPLC-MS/MS data by using Progenesis QI 2.3 (Waters, USA). The preprocessing results generated a data matrix including retention time (R/T), mass to charge ratio (*m*/*z*), and peak intensity. All these data were processed by peak area normalization and 80% rule, and then imported into the software for unsupervised component analysis (principal component analysis PCA) and supervised component analysis (partial least-squared discriminant analysis OPLS-DA) that supplied the intuitive diversity of the different groups. The variable importance in the projection (VIP) score reflects the contribution of the analyzed variables to the OPLS-DA model. Potential differential metabolites were screened according to the criteria of VIP > 1 and *p* < 0.05. The identification and confirmation of these differential metabolites were performed in the human metabolome database (http://www.hmdb.ca/, accessed on 10 September 2022). Based on the KEGG database, the metabolic pathways associated with potential differential metabolites were identified.

### 4.8. Statistical Analysis

SPSS 22.0 statistical software was used for data analysis, and data were expressed as mean ± standard deviation. Independent sample *t*-test was used for comparison between two groups, and one-way analysis of variance was used for comparison between multiple groups. Statistical graphs were generated by GraphPad Prism 8 software, *p* < 0.05 was considered statistically significant.

## 5. Conclusions

Our results indicated that NaB could inhibit AGEs-induced macrophage oxidative stress and inhibit AGEs-induced inflammation via a PI3K-dependent autophagy pathway. Further untargeted metabolomics analysis showed that NaB may inhibit AGEs-induced macrophage inflammation via altering cell metabolisms, which include D-glutamine and D-glutamate metabolism; glycerophospholipid metabolism; biotin metabolism; arginine biosynthesis metabolism; alanine, aspartate, and glutamate metabolism; and sphingolipid metabolism. Our results show a protective effect of butyrate to limit early molecular events underlying inflammation, suggesting a new idea for the prevention and treatment of diabetic complications, such as diabetic nephropathy.

## Figures and Tables

**Table 1:** Trend of differential metabolites of THP-1 cells using *t*-test (*p* < 0.05) and OPLS-DA analysis (VIP > 1).

Compounds	Pathway	*m*/*z*	Retention Time (min)	VIP	*p*-Value	Change
Between AGEs group and NC group						
L-Glutamic acid	D-glutamine and D-glutamate metabolism	344.06	1.11	1.4	0.04	↓
Phosphatidylcholine (PC) (18:3(9Z,12Z,15Z)/18:0)	Glycerophospholipid metabolism	806.57	23.34	1.03	0.04	↓
Biotinyl-5’-AMP	Biotin metabolism	298.57	1.82	1.06	0.03	↑
Oxidized glutathione	Glutathione metabolism	613.16	1.82	1.11	0.02	↑
Ceramide (d18:1/12:0)	Glycerophospholipid metabolism	512.5	21.51	1.15	0.01	↓
Prostaglandin F1a (PGF1a)	Glycerophospholipid metabolism	730.54	21.89	1.06	0.04	↑
N-Methyltryptamine	Tryptophan metabolism	387.19	5.36	1.06	0.02	↑
Elaidic acid	Fatty acid biosynthesis	356.35	5.52	1.09	0.01	↓
Androsterone	Steroid hormone biosynthesis	291.23	7.77	1.15	0	↑
Palmitic acid	Fatty acid biosynthesis	272.26	4.88	1.13	0	↑
13S-hydroxyoctadecadienoic acid	Biosynthesis of unsaturated fatty acids	314.27	4.92	1.18	0	↑
Between AGEs+NaB group and AGEs group						
UDP-N-acetylmuraminate	D-glutamine and D-glutamate metabolism	608.09	0.97	1.49	0.02	↓
Sphingomyelin (SM) (d18:1/16:0)	Sphingolipid metabolism	703.57	23.35	1.61	0.02	↓
Biotinyl-5’-AMP	Biotin metabolism	298.57	1.82	1.59	0	↓
Phosphatidyl ethanolamine (PE)	Glycerophospholipid metabolism	814.63	23.33	1.47	0.03	↑
Thromboxane B2 (TXB2)	Glycerophospholipid metabolism	771.51	23.34	1.54	0.01	↓
L-Glutamic acid	D-glutamine and D-glutamate metabolism	344.06	1.11	1.4	0.04	↑
Undecanoic acid	Fatty acid biosynthesis	390.36	11.81	1.39	0.04	↑
Prostaglandin F1a (PGF1a)	glycerophospholipid metabolism	365.27	9.16	1.47	0.02	↑
phosphatidylcholine (PC)	glycerophospholipid metabolism	482.36	6.96	1.59	0.01	↑

Note: ↑ indicates increase, ↓ indicates decrease, between AGEs group and NC group vs. NC group, between AGEs group and AGEs+NaB group vs. AGEs group.

## Data Availability

The data presented in this study are available on request from the corresponding authors.

## Supplementary Materials

The following supporting information can be downloaded at: https://www.mdpi.com/article/10.3390/molecules27248715/s1, Table S1: The primers used in this experiment.

## Sample Availability

Samples of the compounds are available from the authors.
