# Supplementary material for: Sodium Butyrate Attenuates AGEs-Induced Oxidative Stress and Inflammation by Inhibiting Autophagy and Affecting Cellular Metabolism in THP-1 Cells
Source: Molecules. 2022 Dec 9;27(24):8715. doi: 10.3390/molecules27248715 (PMC9781837; doi:10.3390/molecules27248715)
Supplement: Supplementary file 1 [file molecules-27-08715-s001.zip › molecules-2071048-SI.pdf]

molecules MDPI

Supplementary Materials

Sodium Butyrate Attenuates AGEs-Induced Oxidative Stress and Inflammation by Inhibiting Autophagy and Affecting Cellular Metabolism in THP-1 Cells

Man Yan 1,†, Xiang Li 2,†, Chang Sun 1, Jiajun Tan 1, Yuanyuan Liu 3 , Mengqi Li 1, Zishang Qi 1, Jiayuan He 4, Dongxu Wang 5,* and Liang Wu 1,*
1 Department of Laboratory Medicine, School of Medicine, Jiangsu University, Zhenjiang 212013, China
2 Medical Laboratory Department, Huai'an Second People's Hospital, Huai'an 223022, China
3 Department of Endocrinology, The Affiliated Huai'an No. 1 People's Hospital of NanjingMedical University, Huai'an 223300, China
4 Zhenjiang Center for Disease Control and Prevention, Zhenjiang 212002, China
5 School of Grain Science and Technology, Jiangsu University of Science and Technology,
Zhengjiang 212100, China
* Correspondence: wdx@just.edu.cn (D.W.); wlujs@ujs.edu.cn (L.W.)
† These authors contributed equally to this work.

Table S1. The primers used in this experiment.

Gene	Primer sequences（5'→3'）
GAPDH	F: CATCACTGCCACCCAGAAGACTG<br>R: ATGCCAGTGAGCTTCCCGTTCAG
IL-1β	F: CCTGTCCTGCGTGTTGAAAGA<br>R: GGGAACTGGGCAGACTCAAA
TNF-α	F: AATGGCGTGGAGCTGAGA<br>R: TGGCAGAGAGGAGGTTGAC
NLRP3	F: AACAGCCACCTCACTTCCAG<br>R: CCAACCACAATCTCCGAATG
caspase-1	F: GCACAAGACCTCTGACAGCA<br>R: TTGGGCAGTTCTTGGTATTC
